# Effect of maternal employment on child nutritional status in Bale Robe Town, Ethiopia: a comparative cross-sectional analysis

**DOI:** 10.1017/jns.2022.26

**Published:** 2022-04-18

**Authors:** Bezawit Ketema, Tafese Bosha, Fentaw Wassie Feleke

**Affiliations:** 1 College of Agriculture, Hawassa University, P.O. Box 05, Hawassa, Ethiopia; 2 College of Health Science, Woldia University, P.O. Box 400, Woldia, Ethiopia

**Keywords:** Bale Robe, Ethiopia, Infant and young child, Stunting, Underweight, Wasting, EBF, Exclusive Breastfeeding, HAZ, Height-for-Age *Z*-score, MAD, Minimum Acceptable Diet, MDD, Minimum Diet Diversity, MMF, Minimum Meal Frequency, MUAC, Mid-upper arm circumference, MUACAZ, Mid-upper arm circumference-for-age *Z*-score, PCA, Principal Component Analysis, PI, Principal Investigator, sd, Standard Deviation, SPSS, Statistical Product and Service Solutions, UNICEF, United Nations Children's Fund, WAZ, Weight-for-Age *Z*-score, WHO, World Health Organization, WHZ, Weight-for-Height *Z*-Score

## Abstract

Adequate nutrition is essential for early childhood to ensure healthy growth, proper organ formation, and function, a strong immune system, neurological and cognitive development. The main aim of the present study was to assess the effect of maternal employment on nutritional status among children aged 6–23 months in the town of Bale Robe, Ethiopia. A community-based comparative cross-sectional study was conducted on about 597 (293 unemployed and 304 employed) having children aged 6–23-month-old children sampled were employed with a multistage sampling technique. A face-to-face interview was conducted using a structured pretested questionnaire. Descriptive statistics, binary and multivariable logistic regression analyses were used for the statistical analysis. The magnitude of stunting (39.9 %), underweight (39⋅9 %) and wasting (22⋅2 %) was greater in 6–23-month-old children born to employed mothers than their counterparts in unemployed ones [stunted (31⋅3 %), underweight (24⋅0 %) and wasted (11⋅8 %)]. Being a girl [AOR 0⋅31; 95 % CI (0⋅17, 0⋅54)] in employed mothers and [AOR 0⋅29; 95 % CI (0⋅16, 0⋅51)] in unemployed people significantly protected stunting. This study demonstrated that the nutritional status of 6–23-month-old children is better among unemployed mothers than among employed mothers. Therefore, concerted efforts may decrease child undernutrition in a study area.

## Introduction

Child undernutrition is the most prevalent problem in the world, resulted in both children and the nations^(1)^. Adequate nutrition is essential for early childhood to ensure healthy growth, proper organ formation and function, a strong immune system, neurological and cognitive development^(2)^. Economic growth and human development require well-nourished populations who can learn new skills, think critically and contribute to their communities^(3)^.

Globally, 5⋅4 million children under five (u-5) died before their 5 years and more than 45 % of these deaths were caused by nutrition-related factors^(4)^. The sub-Sahara Africa remains the region with the highest mortality rate for u-5 in the world. For example, 76 deaths/1000 live births^(4)^.

The impact of nutrition on health throughout the course of human life is very profound and is inextricably linked to early childhood cognitive and social development^(5)^. The main effects of undernutrition are believed to occur during the first 2 years of human life^(6,7)^. It is associated with lower educational performance, cognitive deficits and poor economic productivity in adulthood that creates social and economic challenges in disadvantaged communities^(8–10)^. Malnutrition, in all its forms, is a violation of children's right^(2)^.

In Ethiopia, inspiring results in the reduction of the rate of morbidity and mortality of children under 5 years of age, and the initiation of breastfeeding and exclusive breastfeeding (EBF) have made less progress in the past decade^(11,12)^. Children who are currently breastfed decrease from 85 % among children aged 12–17 months to 76 % among children aged 18–23 months. In particular, 6 % of infants under 6 months of age are not breastfed at all^(11)^. This rapid increment of maternal employment has been due to increased household income demand as a result of increased prices of food^(13)^. However, different research studies reported that children of unemployed mothers were at increased risk of developing wasting^(14)^. An inadequate dietary intake and/or disease leads to wasting, which is often a critical nutrition indicator in children u-5 years of age as it predicts child mortality^(15)^. Many factors can cause malnutrition, most of which are related to poor diet or severe and repeated infections, and maternal education^(16)^. Employed mothers are less likely to practice EBF than the unemployed ones^(12,13,17)^. And, non-exclusive breastfeeding has a long-term impact, including poor school performance, reduced productivity, and impaired intellectual and social development^(18)^.

The sustainable development goal targets child health with a mission to end preventable deaths of newborns and children aged 1000 days in 2030, with all countries aiming to reduce neonatal mortality to at least as low as 12 per 1000 live births^(4,9)^. In addition to household responsibilities, many women are now employed outside the home to earn income for their families^(19)^. However, mothers are not still relieved of their customary domestic duties but rather are double burdened with the job and their domestic responsibilities; as primary parent, caregiver and housekeeper. The combination of employment and childcare for women affects not only the work of the mother but also the quality of childcare^(20,21)^. The highest prevalence of malnutrition (wasting) occurs in young children (6–23 months); however, the literature is limited in these population groups. There was no study conducted in this study area previously with this topic. Therefore, evidence of the nutritional status of children aged 6–23 months among employed and unemployed mothers is crucial for further actions in the city of Bale Robe, Oromia Region, Ethiopia.

## Methodology

### Study setting

The present study was conducted in Robe town. This town is located 430 km from Addis Ababa, in the south-east of the country. The altitude is between 2510 and 2800 m above sea level. It receives rain in two seasons, with average downfalls ranging from 800 to 900 mm. The town has three kebeles with total households of 12 883 and a total population of 71 458 (36 015 males and 35 443 females) according to the 2020 projection. Likewise, there are 4452 children aged 6–23 months. In the town, there is one preparatory school, two high schools, thirteen first-cycle primary schools (grades 1–4 elementary school) and thirty-six public and private health facilities.

### Study design and period

A community-based comparative cross-sectional study was conducted in April 2020.

#### Source and study population

The source population for this study was all employed and unemployed mothers who have children aged 6–23 months in the city of Bale Robe. The study population was selected with a simple random group of employed and unemployed mothers from the source population.

#### Inclusion and exclusion criteria

All employed and unemployed mothers who have children aged 6–23 months and live in Bale Robe were eligible for this study. However, mother–child pairs that were severely sick and chronic patients at the time of the survey were not included in this study.

### Sampling

#### Sample size determination

The sample size was calculated using G* power software 3.1.9.7 considering the prevalence of underweight among employed mother–child dyads P2 6⋅3 % and unemployed ones P1 16⋅4^(22)^, design effect 2⋅0 and 5 % non-response rate. The final sample size was 648 (324 employed and 324 unemployed) (Table 1).

**Table 1.** The sample size determination by using G* power software 3.1.9.7 of mother–child dyads aged 6–23 months in Bale Robe Town, Ethiopia, 2020 (*n* 597)

Statistical test: *z*-test proportions difference between two independent proportion type of power analysis: compute required, sample size, given *α*, power and effect size			
Input parameters		Output parameters	
Tail(s)	Two	Critical *z*	1⋅96
Underweight proportion P1 among unemployed mother–child dyads	0⋅164	Sample size group 1	154
Underweight proportion P2 among employed mother–child dyads	0⋅063	Sample size group 2	154
*α* error probability	0⋅05	Total sample size	308
Power (1−*β* error)	0⋅80	Actual power	0⋅80
Allocation ratio N2/N1			
After considering the assumption of design effect 2 and 5 % none response allowance the minimum final sample size was 648 with 324 from employed and 324 unemployed.			

#### Sampling technique and procedures

**Fig. 1.**
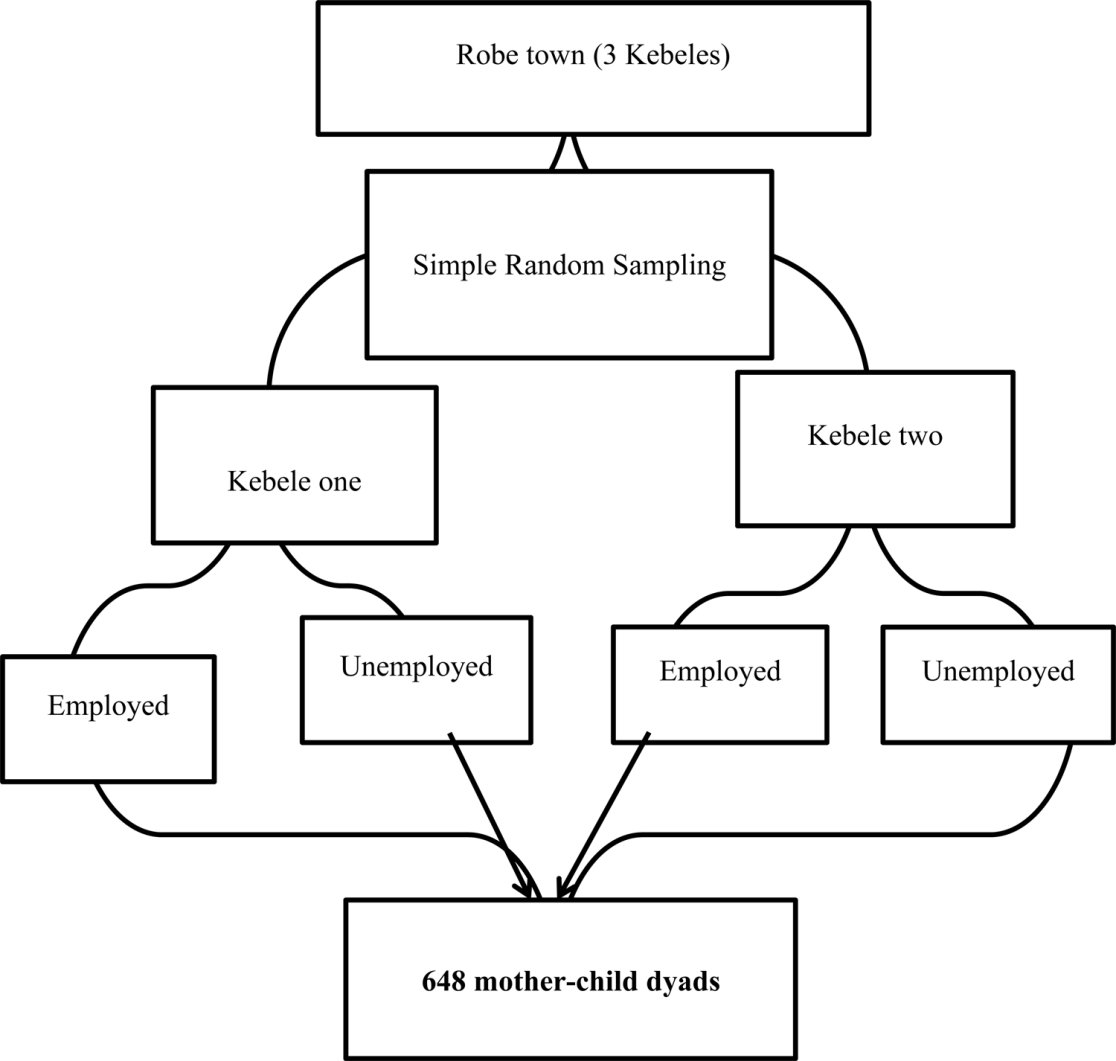
Sampling procedures for selecting 648 mothers for study in Robe town, 2020.

A multistage sampling technique was used. Two kebeles (the lowest administrative unit) were selected from the total of three by simple random sampling. The sample frame was prepared after house-to-house listing of households with children aged 6–23 months among employed and unemployed mothers. The study participants were then randomly selected based on the sampling frame. In households with more than one child aged 6–23 months, one was selected using the lottery method (Fig. 1).

### Data collection

A semi-structured questionnaire was used to collect data on socio-economic and demographic characteristics, healthcare, infant and young child feeding practices (IYCF). The household food security access scale questionnaire was used to assess the state of household food security. Anthropometric measurements were taken by trained data collectors to avoid intra-observer variation using calibrated equipment and standardised techniques. A recumbent length measurement was taken to the nearest 0⋅1 cm using the short height measuring board (short productions, Woonsocket, RI, UK) with the subjects shoeless^(23)^. Weight was measured using a UNICEF Seca electronic personal scale (Seca 881U). Women were asked to remove their children's thick cloth during the measurements. The instrument was calibrated before each measurement. The circumference of the middle of the upper arm was measured with a measuring tape to the nearest to 0⋅1 mm^(24)^. The child's age was collected from the mother. It was confirmed using a birth certificate, a vaccination card and local-events calendar^(23,25)^.

### Data quality assurance

The questionnaire was first developed in English and translated into the local language Afaan Oromo and then translated back to English by language experts to verify its consistency. Data collectors with experience who are fluent in the local language were recruited. The pre-test was conducted by 5 % of the sample size to check the quality of the questionnaire and make appropriate modifications before duplication of the final version on population outside the sampled kebeles.

In order to minimise intra-observer errors, two measurements of height and weight for each child was registered by a single observer, and the third measurement was considered for those cases where the difference between the two measurements was greater than 0⋅5 cm or 0⋅1 kg. The completeness and consistency of the data was checked before the respondent left. Data collection was supervised daily on site by the recruited supervisors and the principal investigator. Extreme values of z-score, >5 or <−5, were excluded from the analysis.

### Data analysis

The data collected was entered into epi data 4.6.2. The data were then exported to SPSS version 25 for analysis. The anthropometric measurements were analysed using WHO Anthro version 3.2.2. The data normality was checked using the Kolmogorov–Smirnov test. Bivariate and multivariate logistic regression analysis was performed to identify the factors associated with the nutritional status of the children. Model fitness was checked using the Hosmer and Lemeshow goodness-of-fit test (*P* > 0⋅05). The odds ratio with a 95 % confidence interval was reported to show the strength of the associations between the outcome and predictor variables. The statistical significance was declared at a *P*-value < 0⋅05. Wealth index was assessed using household assets via principal component analysis adopted from^(26,27)^.

### Ethical approval

This study was conducted according to the guidelines laid down in the Declaration of Helsinki and all procedures involving human subjects/patients were approved by the Institutional Review Board of Hawassa University (Reference number: HU/IRB/215/20; dated: 13 March 2020). Informed (written) consent was obtained from mothers/caregivers with signature/fingerprint all study subjects. A letter of permission was received from the administrative district and zonal health department.

### Operation definition

A child with height/length-for-age (H/LAZ) below −2 standard errors compared with the reference group (*Z*-score < −2) is considered to be stunted^(28)^.

A child with weight-for-age (WAZ) below −2 standard errors compared with the reference group (*Z*-score < −2) is considered to be underweight^(28)^.

A child with weight-for-height/length age (WH/LZ) below −2 standard errors compared with the reference group (*Z*-score < −2) is considered to be wasted^(28)^.

#### Household food insecurity data

Household food insecurity (HFI) was measured using the Household Food Insecurity Access Scale (HFIA), which has nine questions and is related to the households’ experience of food insecurity in the 12 months preceding the survey^(29)^. Then, the HFIA prevalence indicators were categorised households into four^(30)^ levels of HFI: food secure, mild, moderately and severely food insecure. For the present study, only two levels other Household Food Insecurity Access Scale (food secure and insecure) was used because the sample size for this study was small^(31)^.

Maternal employment was defined as the mother's report of whether or not she worked for earnings in the past week. A woman was considered to be an ‘employed’ if she had reported earning income at least three times during the past week weather formal or informal categories, with formal referring to regular wage work, such as governmental or non-governmental organisation employee permanently, and informal referring to street vending, domestic work or vending from a fixed location. A women who have no permanent work or not having any informal work category is considered as unemployed^(32)^.

The minimum acceptable diet is a composite indicator defined as the proportion of children aged 6–23 months who met both minimum dietary diversity and the minimum meal frequency during the previous 24 h^(28)^.

## Results

### Demographic and socio-economic characteristics

The present study was completed with a response rate of 92⋅1 %. Nearly 49⋅0 % and 51⋅0 % of the mothers were employed and unemployed, respectively. At the time of the survey, the majority 93⋅3 % of the respondents were cohabiting in a marriage. Of the mothers who participated in the present study, only 16⋅9 % completed college and higher. Almost 54⋅0 % of the mothers were from male-headed households. Of the households, 33⋅0 % were in the poor wealth quintile. Regarding the children, 67⋅5 % were in the age range of 12–23 months (Table 2).

**Table 2.** Demographic and socio-economic characteristics of mother–child dyads aged 6–23 months in Bale Robe Town, Ethiopia, 2020 (*n* 597)

Variables	Maternal employment status				χ^2^ *P*-value
	Employed (*n* 293)		Unemployed (*n* 304)		
	Number	(%)	Number	(%)	
Maternal age					
19–24	86	(48⋅9)	90	(51⋅1)	0⋅001
25–29	100	(61⋅0)	64	(39⋅0)	
30–34	50	(45⋅0)	61	(55⋅0)	
≥35	57	(31⋅0)	89	(69⋅0)	
Mean ± sd	(28⋅5 ± 6⋅1)		(29⋅7 ± 6⋅8)		
Marital status					
Married	272	(48⋅8)	285	(51⋅2)	0⋅654
Divorced/separated	21	(52⋅5)	19	(47⋅5)	
Maternal education					
Illiterate	0	(0⋅0)	86	(100⋅0)	0⋅000
Primary school (1–8)	93	(42⋅5)	126	(57⋅5)	
Secondary school	105	(55⋅0)	86	(45⋅0)	
College and above	95	(94⋅1)	6	(5⋅9)	
Paternal education					
Illiterate	26	(34⋅2)	50	(65⋅8)	0⋅000
Primary school (1–8)	43	(32⋅1)	91	(67⋅9)	
Secondary school	129	(44⋅8)	159	(55⋅2)	
College and above	95	(96⋅0)	4	(4⋅0)	
Paternal occupation					
Farmer	88	(34⋅9)	164	(65⋅1)	0⋅000
Private worker	46	(92⋅0)	4	(8⋅0)	
Government employee	60	(96⋅8)	2	(3⋅2)	
Merchant	96	(42⋅9)	128	(57⋅1)	
Others^a^	3	(33⋅3)	6	(66⋅7)	
Head of household					
Paternal	139	(42⋅8)	186	(57⋅2)	0⋅003
Maternal	36	(52⋅9)	32	(47⋅1)	
Both	118	(57⋅8)	86	(42⋅2)	
Wealth status					
Poor	90	(45⋅7)	107	(54⋅3)	0⋅217
Medium	96	(47⋅5)	106	(52⋅5)	
Rich	107	(54⋅0)	91	(46⋅0)	
Family size					
≤5	200	(61⋅2)	127	(38⋅8)	0⋅000
>5	93	(34⋅4)	177	(65⋅6)	
Mean ± sd	(4⋅9 ± 1⋅5)		(6⋅0 ± 1⋅4)		
Age (months)					
6–8 months	42	(40⋅0)	63	(60⋅0)	0⋅052
9–11 months	51	(57⋅3)	38	(42⋅7)	
12–23 months	200	(49⋅6)	203	(50⋅4)	
Mean ± sd	(14⋅5 ± 4⋅8)		(14⋅2 ± 5⋅1)		
Sex of child					
Female	142	(46⋅6)	163	(53⋅4)	0⋅208
Male	151	(51⋅7)	141	(48⋅3)	

^a^Daily labour, none government employee.

### Child feeding practices and health-seeking behaviours

We found that all children who participated in the present study had breastfed. Of the sampled children, 38⋅0 % experienced bottle feeding (20⋅3 % employed and 17⋅8 % unemployed mothers). Almost half, 47⋅7 %, of the children were currently being breastfed of which (19⋅4 % employed and 28⋅3 % unemployed). Almost 61⋅0 % of the mothers (35⋅3 % employed and 22⋅6 % unemployed) did not continue breastfeeding for up to 1 year (Table 3).

**Table 3.** Feeding practices of children aged 6–23 months among employed and unemployed mothers in Bale Robe Town, Ethiopia, April 2020 (*n* 597)

Variables	Employed (*n* 293)		Unemployed (*n* 304)		*χ*^2^ *P-*value
	Number	(%)	Number	(%)	
Feeding type					
Bottle feeding	121	(53⋅3)	106	(46⋅7)	0⋅215
Both	121	(47⋅6)	133	(52⋅4)	
Breastfeeding	51	(44⋅0)	65	(56⋅0)	
Exclusive breastfeeding					
No	293	(62⋅6)	175	(37⋅4)	0⋅000
Yes	0	(0⋅0)	129	(100⋅0)	
Currently breastfeeding					
No	177	(56⋅7)	135	(43⋅3)	0⋅000
Yes	116	(40⋅7)	169	(59⋅3)	
Continued breastfeeding at 1 year					
No	211	(57⋅7)	155	(42⋅3)	0⋅000
Yes	82	(35⋅5)	149	(64⋅5)	
Plan breastfeeding till 2 years					
No	232	(48⋅1)	250	(51⋅9)	0⋅429
Yes	54	(52⋅4)	49	(47⋅6)	
Know a right time to start CF					
No	18	(48⋅6)	19	(51⋅4)	0⋅957
Yes	275	(49⋅1)	285	(50⋅9)	
Know the nutritional benefit of CF					
No	8	(50⋅0)	8	(50⋅0)	0⋅940
Yes	285	(49⋅1)	296	(50⋅9)	
Know the amount of CF					
No	37	(46⋅8)	42	(52⋅3)	0⋅669
Yes	256	(49⋅4)	262	(50⋅6)	
Know thickness/consistency of CF					
No	27	(52⋅9)	24	(47⋅1)	0⋅564
Yes	266	(48⋅7)	280	(51⋅3)	
Minimum meal frequency					
Not met	76	(49⋅0)	79	(51⋅0)	0⋅989
Met	217	(49⋅1)	255	(50⋅9)	
Minimum diet diversity					
Not met	48	(56⋅5)	37	(43⋅5)	0⋅141
Met		(47⋅9)		(52⋅1)	
Minimum acceptable diet					
Not met	115	(51⋅3)	109	(48⋅7)	0⋅392
Met	178	(47⋅7)	195	(52⋅3)	
Household food security status					
Insecured	150	(36⋅9)	256	(63⋅1)	0⋅000
Secure	143	(74⋅9)	58	(25⋅1)	

### Nutritional status of children aged 6–23 months

**Fig. 2.**
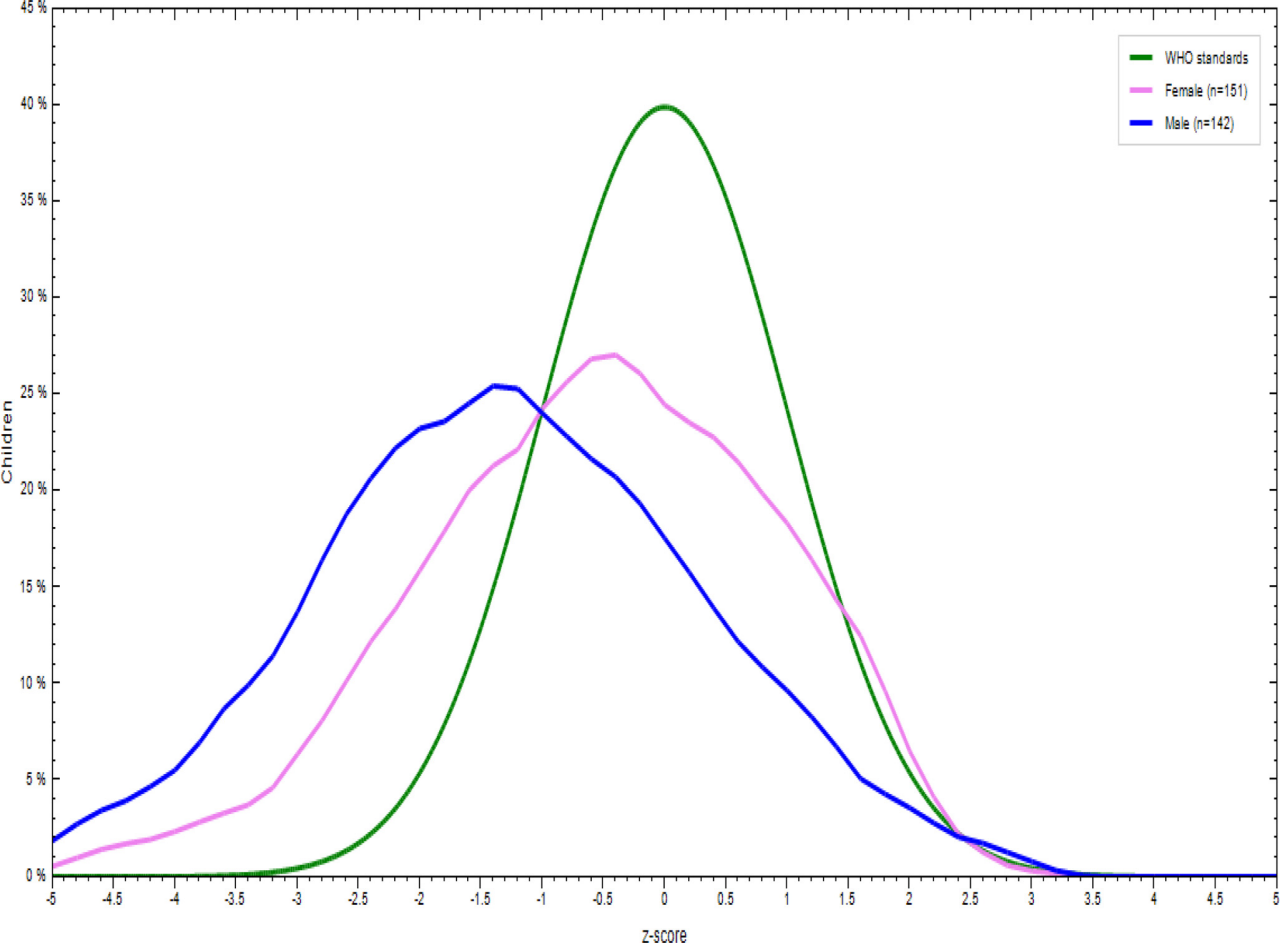
Sex distribution of children aged 6–23 months from employed mothers’ weight-for-length against the WHO standard curve.

**Fig. 3.**
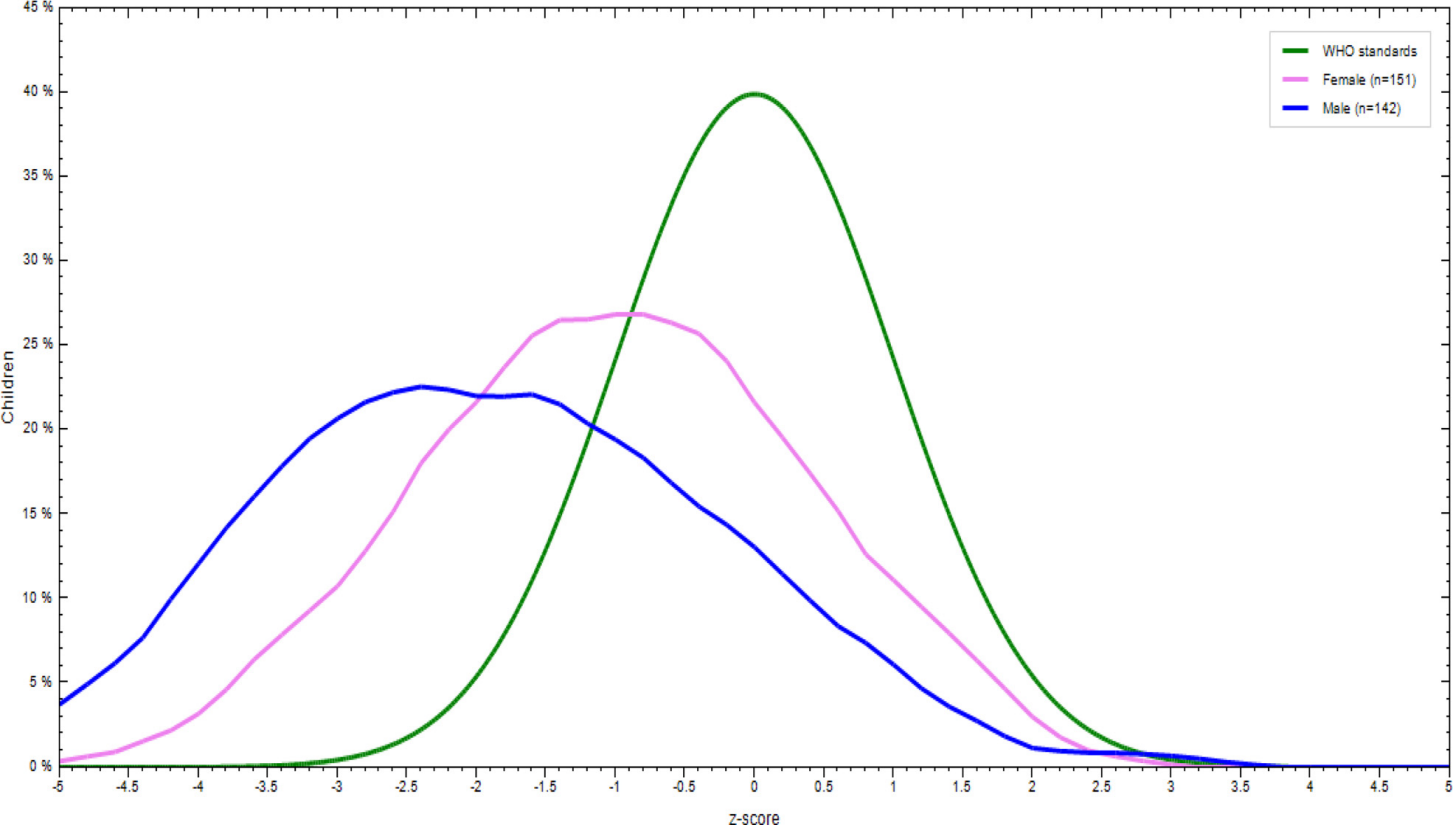
Sex distribution of children aged 6–23 months from employed mothers’ weight-for-age against the WHO standard curve.

**Fig. 4.**
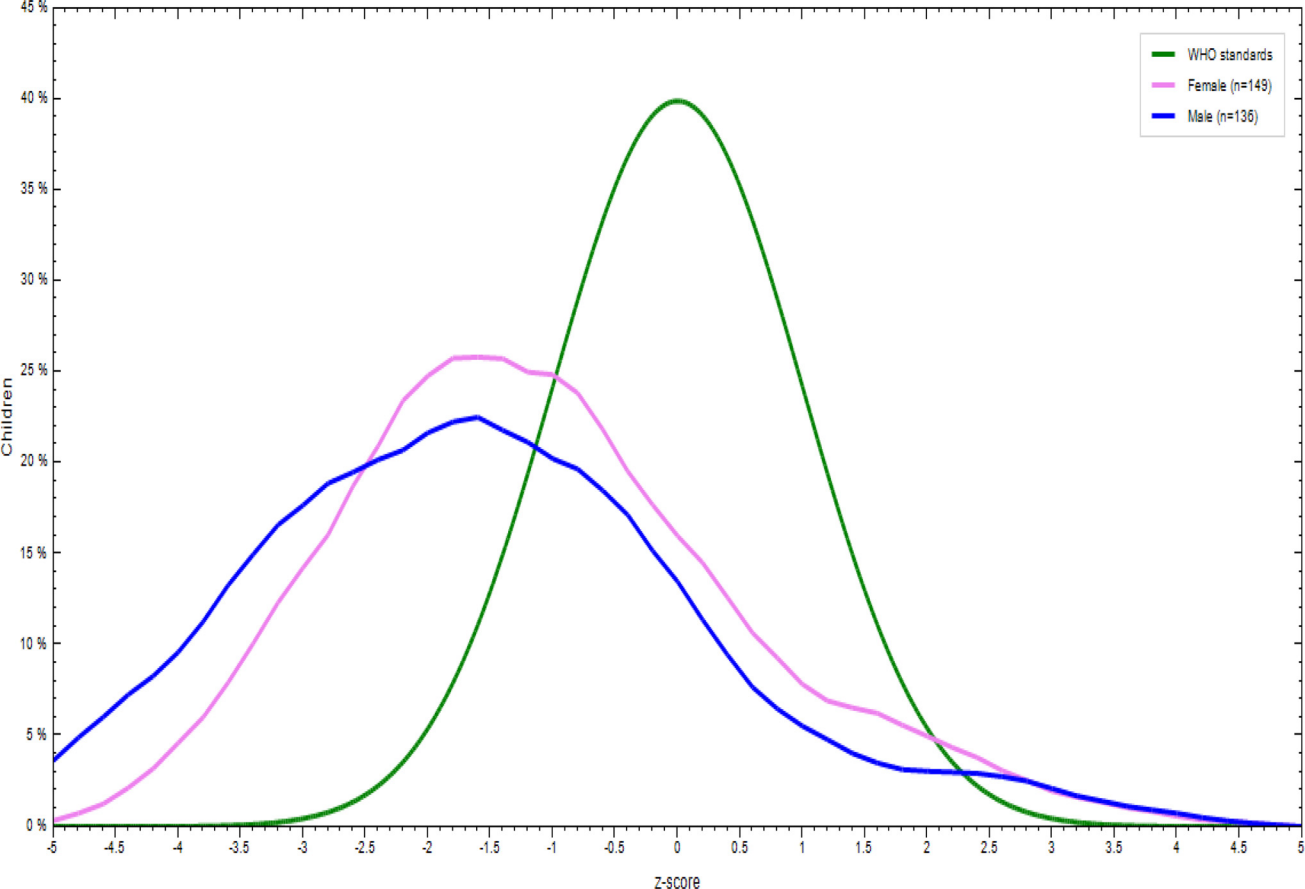
Sex distribution of children aged 6–23 months from employed mothers’ length-for-age against the WHO standard curve.

**Fig. 5.**
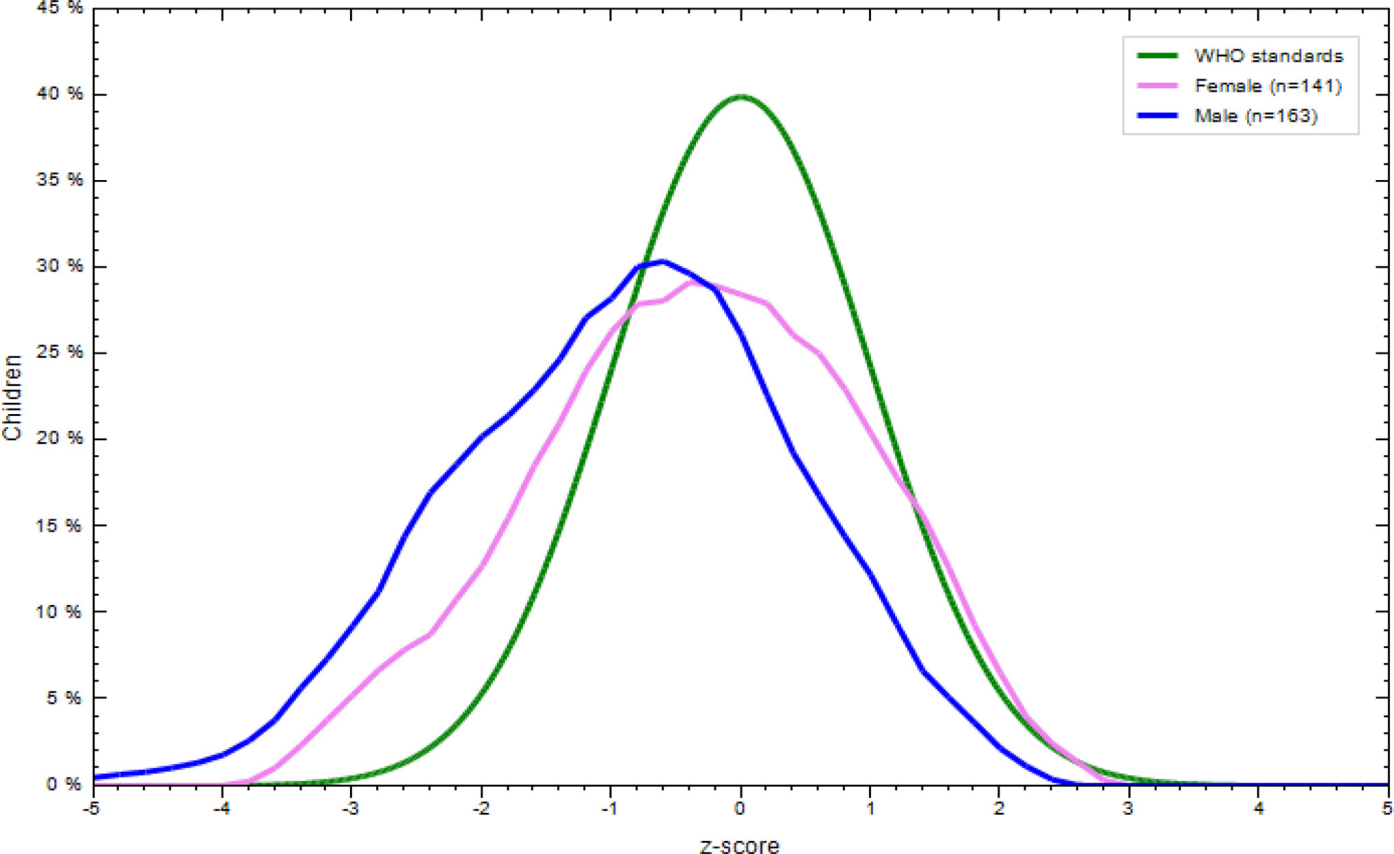
Sex distribution of children aged 6–23 months from unemployed mothers’ weight-for-length against the WHO standard curve.

**Fig. 6.**
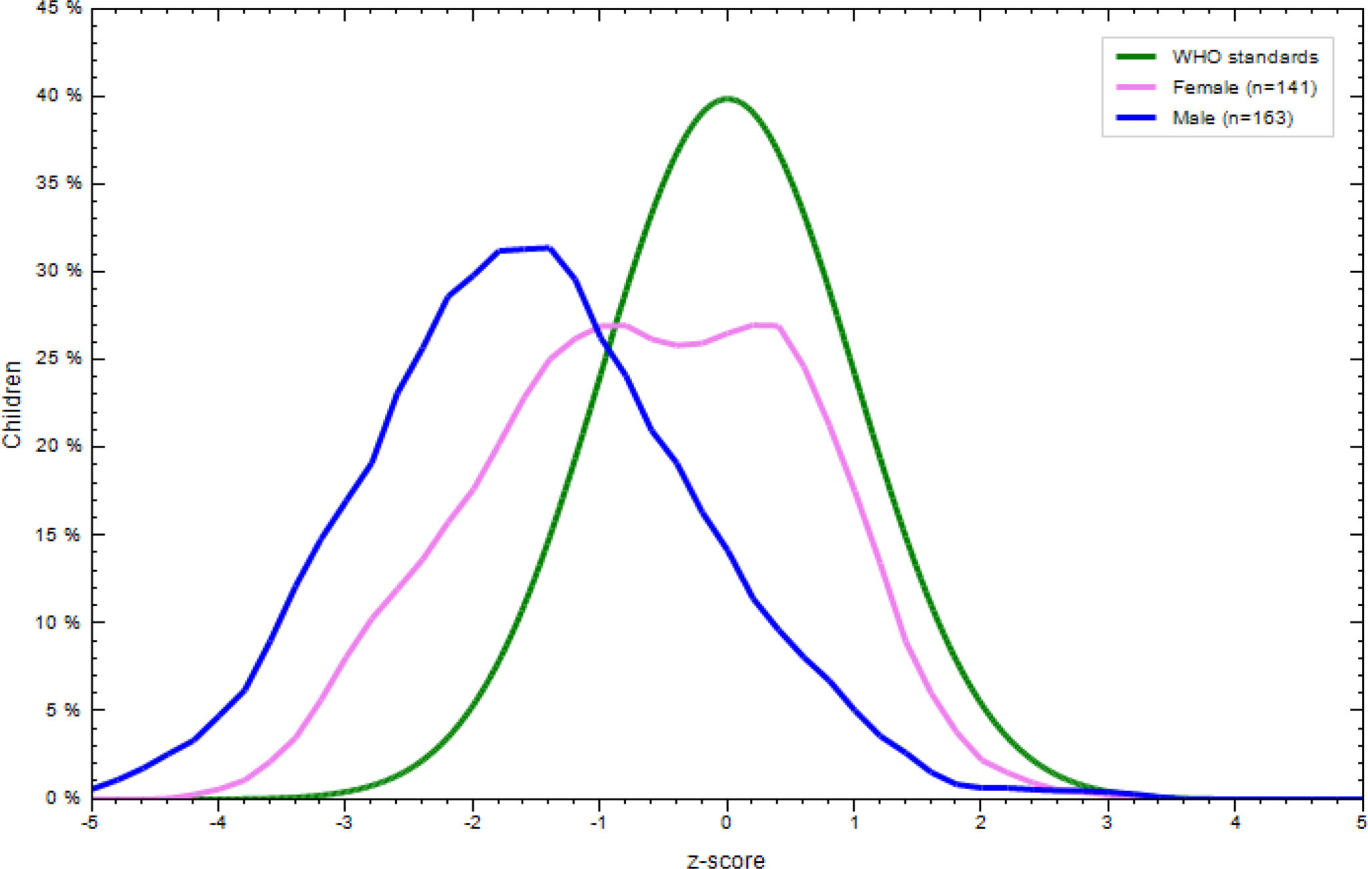
Sex distribution of children aged 6–23 months from unemployed mothers’ weight-for-age against the WHO standard curve.

**Fig. 7.**
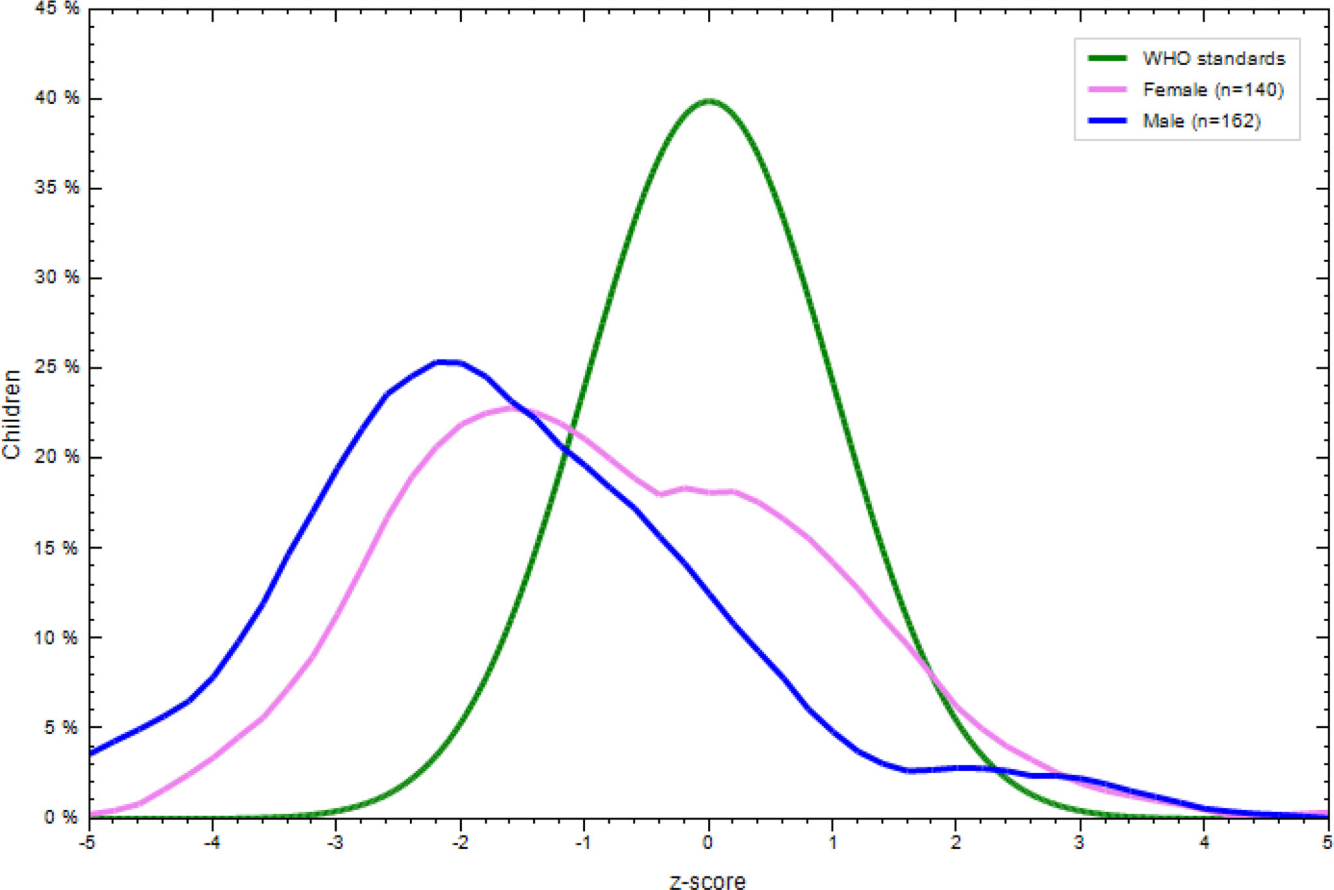
Sex distribution of children aged 6–23 months from unemployed mothers’ length-for-age against the WHO standard curve.

There was a significant difference in the nutritional status of children born to employed and unemployed mothers. More specifically, 31⋅8 % of the children were underweight, with the prevalence tending to be higher in children born to employed mothers. In addition, 16⋅9 % of them were wasted and 35⋅5 % stunted. More boys were stunted, wasted and underweight regardless of the unemployment status of their mothers (Figs. 2–7).

### Factors influencing nutritional status among children aged 6–23 months

After adjusting for confounders, the age of the child and handwashing showed significant associations with stunting. More specifically, being a younger mother (AOR 0⋅32; 95 % CI (0⋅16, 0⋅66)) and (AOR 0⋅32; 95 % CI (0⋅14, 0⋅73)), older child (AOR 3⋅03; 95 % CI (1⋅24, 7⋅42)), being a girl (AOR 0⋅31; 95 % CI (0⋅17, 0⋅54)), medium (AOR 0⋅37; 95 % CI (0⋅19, 0⋅71)) and rich (AOR 0⋅12; 95 % CI (0⋅06, 0⋅24)) wealth status, and household food security (AOR 0⋅42; 95 % CI (0⋅23, 0⋅77)) significantly associated for employed mothers while being older child (AOR 5⋅88; 95 % CI (2⋅37, 14⋅58)), girl (AOR 0⋅29; 95 % CI (0⋅16, 0⋅51)), family size (AOR 0⋅39; 95 % CI (0⋅22, 0⋅69)) and critical handwashing practice (AOR 0⋅31; 95 % CI (0⋅17, 0⋅57)) also significantly predicted stunting (Table 4).

**Table 4.** Predictors of childhood stunting in Bale Robe Town, Ethiopia, 2020 (*n* 597)

Variables	Employed (*n* 293)		COR (95 % CI)	AOR (95 % CI)	*P*-value	Unemployed (*n* 304)		COR (95 % CI)	AOR (95 % CI)	*P*-value
	Not stunted	Stunted				Not stunted	Stunted			
Maternal age										
19–24	43	43	1	1		59	31	1	1	
25–29	68	32	0⋅47(0⋅26–0⋅85)	0⋅32(0⋅16–0⋅66)	0⋅002	45	19	0⋅80(0⋅40–1⋅60)	0⋅76(0⋅35–1⋅67)	0⋅496
30–34	26	24	0⋅92(0⋅46–1⋅85)	0⋅59(0⋅24–1⋅41)	0⋅234	46	15	0⋅62(0⋅30–1⋅28)	0⋅70(0⋅30–1⋅60)	0⋅391
≥35	39	18	0⋅46(0⋅23–0⋅93)	0⋅32(0⋅14–0⋅73)	0⋅007	59	30	0⋅97(0⋅52–1⋅80)	0⋅96(0⋅47–1⋅95)	0⋅905
Age (months)										
6–8	33	9	1	1		56	7	1	1	
9–11	32	19	2⋅18(0⋅86–5⋅52)	1⋅88(0⋅63–5⋅55)	0⋅256	30	8	2⋅13(0⋅71–6⋅45)	1⋅78(0⋅54–5⋅85)	0⋅344
12–23	111	89	2⋅94(1⋅34–6⋅47)	3⋅03(1⋅24–7⋅42)	0⋅015	123	80	5⋅20(2⋅26–1⋅99)	5⋅88(2⋅37–14⋅58)	0⋅000
Sex										
Male	72	79	1	1		80	61	1	1	
Female	104	38	0⋅33(0⋅20–0⋅54)	0⋅31(0⋅17–0⋅54)	0⋅000	129	34	0⋅35(.21–0⋅57)	0⋅29(0⋅16–0⋅51)	0⋅000
Family size										
<5	118	82	1	1		77	50	1	1	
≥5	58	35	0⋅87(0⋅52–1⋅44)	1⋅06(0⋅56–2⋅02)	0⋅848	132	45	0⋅53(0⋅32–0⋅86)	0⋅39(0⋅22–0⋅69)	0⋅001
Wealth status										
Poor	34	56	1	1		71	36	1	1	
Medium	58	38	0⋅40(0⋅22–0⋅72)	0⋅37(0⋅19–0⋅71)	0⋅003	75	31	0⋅82(0⋅46–1⋅46)	0⋅99(0⋅51–1⋅95)	0⋅987
Rich	84	23	0⋅17(0⋅09–0⋅31)	0⋅12(0⋅06–0⋅24)	0⋅000	63	28	0⋅86(0⋅48–1⋅60)	0⋅93(0⋅47–1⋅86)	0⋅841
Critical handwashing time										
Unmet	29	29	1	1		102	70	1	1	
Met	147	88	0⋅60(0⋅34–1⋅07)	0⋅71(0⋅34–1⋅47)	0⋅351	107	25	0⋅34(0⋅20–0⋅58)	0⋅31(0⋅17–0⋅57)	0⋅000
Minimum diet diversity										
Unmet	18	30	1	1		28	9	1	1	
Met	158	87	0⋅33(0⋅17– 0⋅63)	0⋅47(0⋅19–1⋅16)	0⋅100	181	86	1⋅48(0⋅67–3⋅27)	1⋅97(0⋅71–5⋅36)	0⋅187
Minimum acceptable diet										
No	59	56	1	1		75	34	1	1	
Yes	117	61	0⋅55(0⋅34–0⋅89)	0⋅68(0⋅33–1⋅42)	0⋅304	134	61	1⋅00(0⋅61–1⋅67)	0⋅76(0⋅39–1⋅46)	0⋅424
Household food security status										
Secured	81	62	1	1		29	19	1	1	
Insecure	85	55	0⋅76(0⋅47–1⋅21)	0⋅42(0⋅23–0⋅77)	0⋅005	180	76	0⋅64(0⋅34–1⋅22)	0⋅78(0⋅38–1⋅60)	0⋅492

Child sex (AOR 0⋅44; 95 % CI (0⋅22, 0⋅87)), being rich (AOR 0⋅40; 95 % CI (0⋅18, 0⋅90)), handwashing at critical times (AOR 0⋅47; 95 % CI (0⋅22, 0⋅97)), MDD (AOR 0⋅06; 95 % CI (0⋅01, 0⋅40)), household food security (AOR 2⋅94; 95 % CI (1⋅43, 6⋅03)) significantly affected with employed mothers having wasted children aged 6–23 months (Table 5).

**Table 5.** Predictors of childhood wasting in Bale Robe Town, Ethiopia, 2020 (*n* 597)

Variables	Employed		COR (95 % CI)	AOR (95 % CI)	*P*-value	Unemployed		COR (95 % CI)	AOR (95 % CI)	*P*-value
	Not wasted	Wasted				Not wasted	Wasted			
Maternal age										
19–24	67	19	1	1		82	8	1	1	
25–29	77	23	1⋅05(0⋅53–2⋅10)	1⋅14(0⋅50–2⋅61)	0⋅748	54	10	1⋅90(0⋅71–5⋅11)	1⋅71(0⋅61–4⋅81)	0⋅307
30–34	39	11	1⋅00(0⋅43–2⋅31)	0⋅51(0⋅18–1⋅49)	0⋅220	57	4	0⋅72(0⋅21–2⋅50)	0⋅64(0⋅18–2⋅31)	0⋅494
≥35	45	12	1⋅06(0⋅42–2⋅13)	1⋅03(0⋅39–2⋅71)	0⋅956	75	14	1⋅91(0⋅76–4⋅82)	1⋅51(0⋅57–3⋅99)	0⋅406
Sex										
Male	108	43	1	1		119	22	1	1	
Female	120	22	0⋅46(0⋅26–0⋅82)	0⋅44(0⋅22–0⋅87)	0⋅017	149	14	0⋅51(0⋅25–1⋅04)	0⋅49(0⋅23–1⋅03)	0⋅061
Family size										
<5	152	48	1	1		116	11	1	1	
≥5	76	17	0⋅71(0⋅38–1⋅31)	0⋅69(0⋅32–1⋅50)	0⋅349	152	25	1⋅73(0⋅82–3⋅67)	1⋅70(0⋅77–3⋅73)	0⋅189
Wealth status										
Poor	60	30	1	1		93	14	1	1	
Medium	76	20	0⋅06(0⋅27–1⋅02)	0⋅53(0⋅24–1⋅15)	0⋅108	99	7	0⋅47(0⋅18–1⋅22)	0⋅53(0⋅20–1⋅41)	0⋅202
Rich	92	15	0⋅002(0⋅16–0⋅66)	0⋅40(0⋅18–0⋅90)	0⋅027	76	15	1⋅31(0⋅60–2⋅89)	1⋅64(0⋅71–3⋅81)	0⋅249
Handwashing at critical times										
No	33	25	1	1		146	26	1	1	
Yes	195	40	0⋅46(0⋅21–0⋅99)	0⋅47(0⋅22–0⋅97)	0⋅042	122	10	0⋅27(0⋅15–0⋅50)	0⋅45(0⋅20–1⋅02)	0⋅056
Minimum meal frequency										
Unmet	54	22	1	1		69	10	1	1	
Met	174	43	0⋅61(0⋅33–0⋅10)	0⋅19(0⋅03–1⋅37)	0⋅099	199	26	0⋅90(0⋅41–1⋅97)	1⋅07(0⋅11–10⋅76)	0⋅956
Minimum diet diversity										
Unmet	20	28	1	1		31	6	1	1	
Met	208	37	0⋅13(0⋅07–0⋅25)	0⋅06(0⋅01–0⋅40)	0⋅004	237	30	0⋅65(0⋅25–1⋅70)	1⋅59(0⋅14–17⋅83)	0⋅706
Minimum acceptable diet										
Unmet	72	43	1	1		94	15	1	1	
Met	156	22	0⋅25(0⋅13–0⋅42)	3⋅14(0⋅37–26⋅84)	0⋅296	174	21	0⋅76(0⋅0⋅37–1⋅54)	0⋅56(0⋅04–7⋅39)	0⋅660
Household food security status										
Secure	128	15	1	1		39	19	1	1	
Insecure	100	50	4⋅27 (1⋅74–5⋅79)	2⋅94(1⋅43–6⋅03)	0⋅003	229	17	6⋅56(0⋅53–7⋅63)	2⋅21(0⋅70–6⋅94)	0⋅174

Medium (AOR 0⋅35; 95 % CI (0⋅18, 0⋅67)) and rich (AOR 0⋅12; 95 % CI (0⋅06, 0⋅25)) socio-economic status and type of feeding (AOR 0⋅31; 95 % CI (0⋅12, 0⋅80)) were associated with unemployed mothers having underweight children aged 6–23 months. While older age of the child aged 11–23 months (AOR 3⋅58; 95 % CI (1⋅35, 9⋅47)), being girl (AOR 0⋅38; 95 % CI (0⋅21, 0⋅71)), family size (AOR 0⋅50; 95 % CI (0⋅27, 0⋅94)) and handwashing at critical times (AOR 0⋅13; 95 % CI (0⋅06, 0⋅28)) were associated with unemployed mothers having underweight children aged 6–23 months (Table 6).

**Table 6.** Predictors of childhood underweight in Bale Robe Town, Ethiopia, 2020 (*n* 597)

Variables	Employed		COR (95 % CI)	AOR (95 % CI)	*P*-value	Unemployed		COR (95 % CI)	AOR (95 % CI)	*P*-Value
	Not underweight	Underweight				Not underweight	Underweight			
Maternal age										
19–24	50	36	1	1		65	25	1	1	
25–29	65	35	0⋅75(0⋅41–1⋅35)	0⋅64(0⋅32–1⋅30)	0⋅216	48	16	0⋅87(0⋅42–1⋅80)	0⋅81(0⋅35–1⋅90)	0⋅633
30–34	24	26	1⋅51(0⋅75–3⋅03)	1⋅10(0⋅46–2⋅62)	0⋅831	51	10	0⋅51(0⋅23–1⋅16)	0⋅49(0⋅19–1⋅24)	0⋅133
≥35	37	20	0⋅75(0⋅38–1⋅50)	0⋅66(0⋅29–1⋅47)	0⋅306	67	22	0⋅85(0⋅44–1⋅66)	0⋅74(0⋅34–1⋅60)	0⋅44
Age of child (months)										
6–8	25	17	1	1		57	6	1	1	
9–11	29	22	1⋅12(0⋅49–2⋅56)	0⋅77(0⋅29–2⋅20)	0⋅589	31	7	2⋅15(0⋅66–6⋅95)	1⋅22(0⋅34–4⋅39)	0⋅757
12–23	122	78	0⋅94(0⋅48–1⋅85)	0⋅62(0⋅29–2⋅02)	0⋅225	143	60	3⋅99(1⋅63–9⋅74)	3⋅58(1⋅35–9⋅47)	0⋅010
Sex of child										
Male	81	70	1	1		96	45	1	1	
Female	95	47	0⋅57(0⋅36–0⋅92)	0⋅62(0⋅28–1⋅35)	0⋅049	135	28	0⋅44(0⋅26–0⋅76)	0⋅38(0⋅21–0⋅71)	0⋅002
Family size										
Less than five	120	80	1	1		91	36	1	1	
Five and above	56	37	0⋅99(0⋅60–1⋅64)	1⋅21(0⋅63–2⋅31)	0⋅564	140	37	0⋅67(0⋅39–1⋅13)	0⋅50(0⋅27–0⋅94)	0⋅03
Wealth status										
Poor	31	59	1	1		80	27	1	1	
Medium	59	37	0⋅33(0⋅18–0⋅60)	0⋅35(0⋅18–0⋅67)	0⋅002	84	22	0⋅78(0⋅41–1⋅47)	0⋅97(0⋅46–2⋅06)	0⋅947
Rich	86	21	0⋅13(0⋅07–0⋅25)	0⋅12(0⋅06–0⋅25)	0⋅000	67	24	1⋅06(0⋅56–2⋅01)	1⋅40(0⋅67–2⋅94)	0⋅375
Handwashing at critical times										
No	27	31	1	1		110	62	1	1	
Yes	149	86	0⋅50(0⋅28–0⋅90)	0⋅66(0⋅32–1⋅37)		121	11	0⋅16(0⋅08–0⋅32)	0⋅13(0⋅06–0⋅28)	0⋅000
Feeding type										
Bottle feeding	72	49	1	1		82	24	1	1	
Both	67	54	1⋅18(0⋅71–1⋅97)	1⋅25(0⋅69–2⋅28)	0⋅469	85	38	1⋅37(0⋅76–2⋅47)	1⋅59(0⋅81–3⋅12)	0⋅195
Breastfeeding	37	14	0⋅56(0⋅27–1⋅14)	0⋅31(0⋅12–0⋅80)	0⋅015	54	11	0⋅70(0⋅32–1⋅54)	0⋅76(0⋅29–2⋅00)	0⋅180
Minimum meal frequency										
Unmet	40	36	1	1		59	20	1	1	
Met	136	81	0⋅66(0⋅39–1⋅12)	0⋅56(0⋅08–4⋅18)	0⋅573	172	53	0⋅91(0⋅50–1⋅65)	3⋅41(0⋅31–38⋅18)	0⋅320
Minimum diet diversity										
Unmet	15	33	1	1		28	9	1	1	
Met	161	84	0⋅24(0⋅12–0⋅46)	0⋅15(0⋅02–1⋅06)	0⋅058	203	64	0⋅98(0⋅44–2⋅19)	2⋅15(0⋅19–23⋅84)	0⋅533
Minimum acceptable diet										
Unmet	53	62	1	1		81	28	1	1	
Met	123	55	0⋅38(0⋅24–0⋅62)	1⋅33(0⋅16–11⋅26)	0⋅796	150	45	0⋅87(0⋅50–1⋅50)	0⋅30(0⋅02–3⋅77)	0⋅349
Household food security status										
Secure	93	50	1	1		37	11	1	1	
Insecure	83	67	1⋅50(0⋅94–2⋅40)	1⋅14(0⋅64–2⋅04)	0⋅640	194	62	1⋅08(0⋅52–2⋅23)	1⋅79(0⋅79–4⋅09)	0⋅164

## Discussion

The present study revealed that 6–23 months of children born to employed mothers had a higher chance of undernutrition (stunting, wasting and underweight) in Bale Robe Town. The present study revealed that more than half (39⋅9 %) of children of employed mothers were stunted compared to unemployed (31⋅3 %). Similarly, the chance of a child being underweight is also significantly higher in the case of employed mothers (39⋅9 %) than in their counterparts belonging to unemployed ones (24⋅0 %). In the same way, wasting is lower among children of unemployed mothers (11⋅8 %) than among employed ones (22⋅2 %). This could happen because mothers cannot provide the care the child needs, which in return can lead to infections and malnutrition due to the effect of access to water, sanitation and handwashing facilities in Ethiopia^(33)^. Furthermore, malnourished children will suffer from poor physical and cognitive development, as well as low school performance. A previous research study in Adama city reported supportive findings on stunting (33⋅8 %), underweight (12⋅6 %) and wasting (8⋅3 %)^(34)^. Furthermore, our finding is consistent with previous reports on the effect of maternal employment on child nutritional status in Sodo town, Wolayta, Southern Ethiopia^(22)^, and Nigeria^(35)^ and Sri Lanka from abroad^(36)^. Furthermore, the prevalence of wasting and underweight in the present study area is comparable with the ones reported from Nigeria^(37)^.

Employed younger maternal age with children aged 6–23 months was predictor of child stunting compared to their counterparts. This finding is evidently supported by a study conducted in Ghana reported that children of teenage mothers, compared to those of adult mothers, were eight times more likely to be stunted, three times more likely to be wasted and thirteen times more likely to be underweight after adjusting for potential confounders^(38)^. This could also be due to the association of maternal health literacy with early childhood nutritional status which is evidenced in India^(39)^ and maternal depression in Pakistan^(40)^.

The result of multivariate analysis showed that the probability of being stunted was 63 % protective among children from rich families compared to those from poor families. This finding is consistent with studies revealed in Ethiopia, Sri Lanka and Tanzania^(41–45)^. The possible explanation might be related to the negative effect of poor socio-economic status on access to household food, health service utilisation, availability of improved water sources and sanitation facilities^(42)^.

The odds of stunting were higher among children aged 12–23 months compared to those aged 6–11 months for employed and unemployed mothers. The odds of being underweight were also higher among children aged 12–23 months for unemployed mothers compared to 6–11 months. This is consistent with the finding from Dabat district, Northern Ethiopia^(42)^. Furthermore, the present result agrees with that of the Central African Republic in which poor growth of children was correlated with the old age of children^(46)^. This could also be evidenced by maternal undernutrition^(47)^.

The present study demonstrated that being a boy is associated with stunting (for both employed and unemployed mothers), underweight (employed mothers) and waste (unemployed mothers). This result is different from the one reported from Ethiopia such as Somali Region^(48)^ and West Guji Oromia region^(49)^, Egypt^(50)^, Pakistan^(51)^ and South Asia^(52)^. This could be attributable to male patriotism in some communities where girls are less socially and nutritionally favoured. The present study was in line with studies conducted in Ethiopia such as Mekelle City, Tehuledere district^(53)^, Jimma^(54)^, Chiro town^(55)^ and Wukro town^(56)^ which confirmed that males were more affected due to thinness than girls. This variable includes several African countries including Pakistan^(57)^. This could be due to the variation in maturation time in boys and girls, for which girls reached maturation earlier than boys.

Studies in other developing countries also claimed that stunting was less common in early childhood as they were on breastfeeding^(42,58)^; however, because of inappropriate complementary feeding practice and higher nutritional demand, the risk of impaired linear growth increases as the child's age advances^(59)^. For example, employed mothers stopped breastfeeding and introduced supplements and breast milk substitutes earlier than unemployed ones in Uganda, USA and France^(60–62)^. A possible explanation for this is that, apart from household responsibilities, mothers are employed outside their home to earn income for their families^(19)^ and this, in turn, makes them too busy to provide adequate time for their child feeding and caring. Therefore, children born to employed mothers face suboptimal breastfeeding, and earlier introduction of complementary feeding, and receive less social care. However, the employment of mothers has increased rapidly due to an increased demand for household income as a result of increased prices of food^(13)^. Hygiene and sanitation practices could have a significant contribution to preventing child infection, which is the second wing of the immediate causes of child malnutrition^(63,64)^.

Furthermore, the present study found that food insecurity in households in children born to employed mothers was associated with stunting and underweight. These results are consistent with the previous ones from East Badawacho district^(65)^, Butajira^(66)^ and Dabat^(67)^ of Ethiopia. Furthermore, children of employed mothers from poor households were more likely to be stunted, and underweight^(68)^. This is supported by studies from Hossana town, Ethiopia^(69)^, Tanzania^(70)^, and Northern Ghana^(71)^, and Nepal^(72,73)^. This could be because household food insecurity and child undernutrition are critical problems in the present study setting. Socio-demographic factors, poor childcare practices, infection and food insecurity had a positive association with children undernutrition^(74)^.

### Strengths and limitations of the study

The study is mentioned as first baseline for the study locality. However, there may be recall bias in child feeding practices. Furthermore, there could be a social desirability bias in terms of the socio-economic status of the respondents.

## Conclusion

The present study showed that the nutritional status of 6–23-month-old children is better among unemployed mothers than among employed mothers. Furthermore, this demonstrated that poverty had an effect on stunting, wasting and underweight in children born from employed mothers. In addition, being a boy was significantly associated with stunting and wasting among employed mothers while underweight among unemployed once. Concerted efforts could reduce the number of children under 6 months of age undernutrition in the study location.
